# Micro CT Analysis of Spine Architecture in a Mouse Model of Scoliosis

**DOI:** 10.3389/fendo.2015.00038

**Published:** 2015-03-19

**Authors:** Chan Gao, Brian P. Chen, Michael B. Sullivan, Jasmine Hui, Jean A. Ouellet, Janet E. Henderson, Neil Saran

**Affiliations:** ^1^Bone Engineering Labs, Research Institute-McGill University Health Centre, Montreal, QC, Canada; ^2^Department of Medicine, McGill University, Montreal, QC, Canada; ^3^Department of Kinesiology and Physical Education, McGill University, Montreal, QC, Canada; ^4^Biotechnology Program, University of British Columbia, Burnaby, BC, Canada; ^5^Department of Surgery, McGill University, Montreal, QC, Canada

**Keywords:** progressive kyphoscoliosis, fibroblast growth factor receptor 3 deficiency, parathyroid hormone-related peptide treatment, radiography, micro-computed tomography, histology

## Abstract

**Objective:** Mice homozygous for targeted deletion of the gene encoding fibroblast growth factor receptor 3 (FGFR3^−/−^) develop kyphoscoliosis by 2 months of age. The first objective of this study was to use high resolution X-ray to characterize curve progression *in vivo* and micro CT to quantify spine architecture *ex vivo* in FGFR3^−/−^ mice. The second objective was to determine if slow release of the bone anabolic peptide parathyroid hormone related protein (PTHrP-1-34) from a pellet placed adjacent to the thoracic spine could inhibit progressive kyphoscoliosis.

**Materials and methods:** Pellets loaded with placebo or PTHrP-1-34 were implanted adjacent to the thoracic spine of 1-month-old FGFR3^−/−^ mice obtained from in house breeding. X rays were captured at monthly intervals up to 4 months to quantify curve progression using the Cobb method. High resolution post-mortem scans of FGFR3^−/−^ and FGFR3^+/+^ spines, from C5/6 to L4/5, were captured to evaluate the 3D structure, rotation, and micro-architecture of the affected vertebrae. Un-decalcified and decalcified histology were performed on the apical and adjacent vertebrae of FGFR3^−/−^ spines, and the corresponding vertebrae from FGFR3^+/+^ spines.

**Results:** The mean Cobb angle was significantly greater at all ages in FGFR3^−/−^ mice compared with wild type mice and appeared to stabilize around skeletal maturity at 4 months. 3D reconstructions of the thoracic spine of 4-month-old FGFR3^−/−^ mice treated with PTHrP-1-34 revealed correction of left/right asymmetry, vertebral rotation, and lateral displacement compared with mice treated with placebo. Histologic analysis of the apical vertebrae confirmed correction of the asymmetry in PTHrP-1-34 treated mice, in the absence of any change in bone volume, and a significant reduction in the wedging of intervertebral disks (IVD) seen in placebo treated mice.

**Conclusion:** Local treatment of the thoracic spine of juvenile FGFR3^−/−^ mice with a bone anabolic agent inhibited progression of scoliosis, but with little impact on kyphosis. The significant improvement in IVD integrity suggests PTHrP-1-34 might also be considered as a therapeutic agent for degenerative disk disorders.

## Introduction

Scoliosis is a pathological condition characterized by lateral curvature of the spine in the coronal (front–back) plane, whereas kyphosis is excessive angulation of the spine in the sagittal (sideways) plane. Scoliotic disorders are generally classified into congenital forms, such as those arising from fundamental flaws in skeletal patterning in the embryo, those resulting from neuromuscular disorders, and the vast majority of cases that are of undetermined origin (1, 2). The presence of scoliosis before the age of 5 years is defined as early onset scoliosis (EOS) and leads to severe, three dimensional deformation of the spine in ~1/2500 children. The deformity impedes normal lung development and contributes to the primary morbidity associated with the condition (3). While the effects of EOS treatment on morbidity are not fully understood, it has been established that early fusion of the spine to prevent progression of the curve can be further detrimental to pulmonary health (4). Alternative growth-sparing techniques that allow ongoing development of the spine and thoracic cage are therefore encouraged. These techniques rely on bracing, serial casting, and in refractory cases the use of “growing rods” for bio-mechanical correction and inhibition of curve progression. Growing rods are surgical implants placed in the posterior spine to guide longitudinal growth and alignment of the spine. The treatment is fraught with serious complications associated with high financial and psychosocial burden that include significant pain, loss of spinal mobility, and junctional deformity in adjacent vertebrae.

The vast majority of pre-clinical animal models that have been developed to study scoliosis have relied on post-natal interventions including damage to the central nervous system and biomechanical constraints on spine growth (5). However, a genetic basis for some forms of scoliosis is suggested by studies of mice from dams subjected to teratogens like *N*-ethyl-*N*-nitrosourea (6, 7) and carbon monoxide (8), as well as those carrying spontaneous or targeted mutations in genes known to impact the development of neuromuscular and skeletal tissues. For example, mice homozygous for targeted disruption of the gene encoding fibroblast growth factor receptor 3 (*FGFR3* OMIM 134934) exhibit EOS in association with overgrowth of the axial and appendicular skeleton (9, 10). Skeletally mature FGFR3^−/−^ mice have also been characterized with reduced cortical bone thickness, defective trabecular bone mineralization, premature joint degeneration, and early arthritis (11, 12).

Parathyroid hormone related protein (PTHrP) was first cloned from malignant tumors removed from patients with a para-neoplastic syndrome known as humoral hypercalcemia of malignancy (13, 14). When released by the tumor into the circulation, the protein, which shares sequence homology with parathyroid hormone (PTH), activates PTH receptors to promote release of calcium from bone and its retention in the kidney (15, 16). Biochemical and physiological analyses determined that PTHrP-1-34 is homologous with PTH-1-34 and binds to the PTH cell surface receptor on target cells, including chondrocytes and osteoblasts (17, 18). A central role for PTHrP in skeletal development was later demonstrated when PTHrP-null mice died at birth with lethal skeletal dysplasia (19) and their heterozygote littermates developed osteopenia as young adults (20). Unlike the catabolic effect mediated by high levels of circulating PTH/PTHrP, the 1–34 peptide mediates anabolic activity on cells when released into the bone micro-environment (21).

FGFR3^−/−^ mice have been characterized with osteopenia (11), which has been established as a risk factor for curve progression in patients with scoliosis (22). The objective of this study was to use micro CT to quantify progressive kyphoscoliosis in FGFR3^−/−^ mice and to determine if treatment with PTHrP-1-34 could inhibit curve progression. Slow release of the bone anabolic peptide adjacent to the thoracic spine inhibited progression of scoliosis but had little impact on kyphosis or bone volume. The micro CT data was supported by histological analyses of the spine.

## Materials and Methods

### *In vivo* radiologic imaging of mouse spine

All *in vivo* procedures were conducted as outlined in a protocol approved by McGill Facility Animal Care Committee in compliance with the Canadian Council on Animal Care. Male and female FGFR3^+/+^ and FGFR3^−/−^ mice used for this study were obtained through in house breeding from a colony maintained for 20 generations on a C3H background. The mice were lightly anesthetized at the indicated times and carefully positioned to capture coronal and sagittal plane radiographs (Kubtek XPERT 80, Milford, CT, USA). Gingko CADx 2.4.1 software (Valladolid, Spain) was used to assess gross anatomical measurements and to measure the magnitude of the largest scoliotic and thoraco-lumbar kyphotic curves, according to the Cobb method (23).

### Treatment with synthetic PTHrP-1-34

Slow-release pellets measuring 3 mm in diameter and designed to release 1.0 mg PTHrP-1-34 (*N* = 16) or placebo (*N* = 18) over 60 days were manufactured by Innovative Research of America (Sarasota, FL, USA). The pellets were implanted in 4-week-old FGFR3^−/−^ mice under general anesthesia on the left side of the spine adjacent to the thoraco-lumbar junction, then maintained for 12 weeks with free access to food and water. Radiographic imaging on anesthetized mice was performed at monthly intervals as described above, and the mice were euthanized by injection of an overdose of anesthetic at 16 weeks of age. Spines were extracted by cutting through the neck and separating the thoracic and lumbar vertebrae from the ribs and pelvis, cleaned of soft tissue, and fixed in 4% paraformaldehyde for 24 h. The fixed spines were then washed in several changes of sterile PBS and stored in PBS at 4°C until the time of micro CT analysis (24).

### Post-mortem micro CT analyses

High resolution scans of the spine of FGFR3^−/−^ (*N* = 16 treated with PTHrP-1-34; *N* = 18 treated with placebo) and FGFR3^+/+^ (*N* = 5) mice from C5/6 to L4/5 were captured to evaluate the 3D structure, as well as the rotation of the vertebrae in the curve of FGFR3^−/−^ mice. Scans were captured at 8 μm spatial resolution on a Skyscan 1172 micro-computed tomograph (micro CT, Bruker, Kontich, Belgium) equipped with a 0.5 mm aluminum filter at a voltage of 55 kV, a current of 180 μA, and a power source of 10 W. The rotation step size used for acquiring images was 0.4°. NRecon and CTVol software was used for transverse 2D cross-sectional reconstructions and 3D reconstruction of sub-sets of images.

CTAn software supplied with the instrument was used for quantitative analysis of bone mass and architecture, apical vertebra axial rotation, and geometric properties. Axial rotation of the apical vertebra, as defined by the Scoliosis Research Society (25), was quantified by loading the dataset in Dataviewer and rotating the images to align the anterior and posterior borders of the L5 vertebral body in the horizontal plane. The fifth lumbar vertebra then served as the reference point for measuring the angle of rotation. The properly aligned images were saved as a new dataset and loaded into CTAn to locate the apical vertebra, which is the most rotated vertebra (26). The angle of rotation was measured from the horizontal plane to the straight line from the mid-points of the anterior to posterior surfaces of the apical vertebra.

The L5 vertebral body lays outside of the scoliotic curve in all animals and was therefore used as a reference point to assess bone volume/total volume (BV/TV), which is the primary measure of bone quality. The transverse view image datasets for L5 from FGFR3^−/−^ and FGFR3^+/+^ mice were used to isolate trabecular bone as the ROI by excluding the vertebral arch and cortical bone. Trabecular bone was then segmented into left, middle, and right portions to calculate BV/TV of the outer (left and right) segments independently. A 3D volume of interest (VOI) that included only the middle 50% of the vertebral body was selected to avoid the endplate region. BV/TV values for bone within the VOI was obtained using CTAn. BV/TV for the apical vertebra in FGFR3^−/−^ mice was calculated in the same manner.

The concave/convex geometry of the apical vertebra in FGFR3^−/−^ spines was evaluated using cross-sectional images isolated in CTAn and loaded for 3D viewing in Dataviewer. The mid-vertebral body coronal image was saved and the lengths of the concave and convex sides measured from the superior to inferior endplates using CTAn to quantify lateral asymmetry. The same measurements were made on the apical-equivalent vertebra in FGFR3^+/+^ mice as a control.

### Histological analyses

After micro CT analyses, the apical and adjacent vertebrae of FGFR3^−/−^ (*N* = 16 treated with PTHrP-1-34; *N* = 18 treated with placebo) spines, and the corresponding vertebrae from the FGFR3^+/+^ (*N* = 5) spines, were carefully dissected free from the remaining vertebrae, fixed in 4% paraformaldehyde for 24 h, and embedded at low temperature in poly-methyl-methacrylate (PMMA) as described previously (24). Five micron sections were cut from the polymerized blocks on a Leica microtome and stained with von Kossa/Toluidine blue to distinguish mineralized from soft tissue, with Safranin O to delineate intervertebral disks (IVDs) and vertebral end plates, and for alkaline phosphatase (ALP) and tartrate-resistant acid phosphatase (TRAP) in anabolic and catabolic cells, respectively. Image J software was used to quantify ALP and TRAP activity on the concave vs convex aspects.

### Statistical analyses

A Mann–Whitney *U* test for unpaired samples in the IBM SPSS Statistics (Armonk, NJ, USA) package was used to compare differences in the degrees of scoliosis, kyphosis, axial rotation of apical vertebrae, and concave vs convex height of the apical vertebral body. A one way analysis of variance (ANOVA) with *post hoc* Tukey’s test was used for comparison of BV/TV. Differences were considered significant at *p* < 0.05.

## Results

Kyphoscoliosis was previously noted in juvenile FGFR3^−/−^ mice on a mixed C57Bl6 background but was never characterized (9, 10). The genotype was later transferred onto a C3H background to improve longevity and enable phenotyping of adult mice (11, 12). Table 1 shows the Cobb angle of thoracic vertebrae measured in large cohorts of FGFR3^+/+^ and FGFR3^−/−^ mice, aged between 1 and 6 months, that were euthanized in our laboratory. Intra-group variations in spine curvature are remarkably small amongst FGFR3^+/+^ mice compared with the large variations in groups of FGFR3^−/−^ mice. Beyond 1 month, the mean Cobb angle is significantly greater at all ages in mutant FGFR3^−/−^ mice compared with FGFR3^+/+^ wild type mice (33.30 ± 19.55° vs 5.06 ± 3.39° at 4 months, *p* < 0.01) and appears to stabilize around 4 months of age when the animals reach skeletal maturity.

**Table 1:** **Development of scoliosis, measured by Cobb angle, in growing FGFR3^−/−^ mice**.

Genotype	Age (months)	*N*	Minimum (degrees)	Maximum (degrees)	Mean ± SD (degrees)
FGFR3^+/+^	1	9	0.37	14.41	6.2 ± 4.64
FGFR3^−/−^	1	12	1.15	46.60	11.01 ± 11.95
FGFR3^+/+^	2	25	1.43	15.04	4.86 ± 3.74
FGFR3^−/−^	2	40	0.91	62.46	15.79 ± 12.82*
FGFR3^+/+^	3	36	0.65	14.98	4.91 ± 2.92
FGFR3^−/−^	3	57	2.71	105.47	31.60 ± 21.33*
FGFR3^+/+^	4	38	0.95	14.76	5.06 ± 3.39
FGFR3^−/−^	4	43	5.51	101.28	33.30 ± 19.55*
FGFR3^+/+^	5	16	0.93	13.19	5.23 ± 3.31
FGFR3^−/−^	5	20	6.98	80.18	32.45 ± 19.29*
FGFR3^+/+^	6	17	0.93	11.91	4.93 ± 3.55
FGFR3^−/−^	6	21	15.29	77.34	40.92 ± 17.14*

Significantly different from FGFR3^+/+^ mice *p < 0.01.

### Progression of scoliosis in FGFR3^−/−^ mice

**Figure 1:**
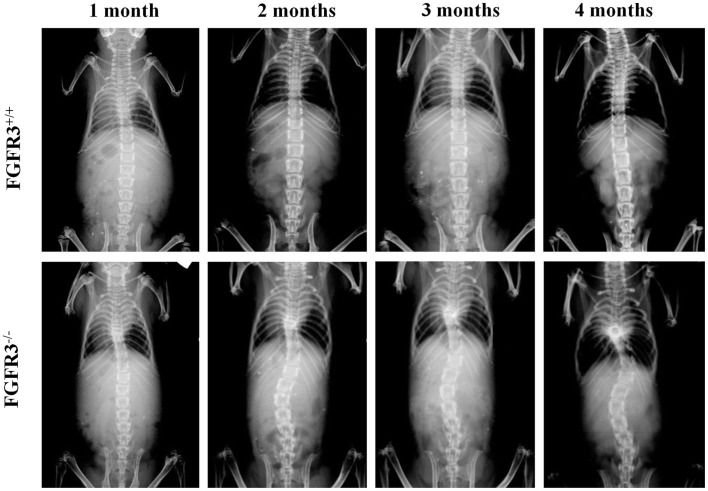
**Radiographic evidence of progressive scoliosis in FGFR3^−/−^ mice: high resolution radiographs were taken of anesthetized mice at monthly intervals up to 4 months of age**. Serial radiographs of representative mice shows no evidence of spine curvature in the FGFR3^+/+^ animal (upper) at any time point. The FGFR3^−/−^ animal (lower) shows little evidence of deformity at 1 month, compared with the sigmoid curve that is evident at 2 months, and increases in severity to 4 months.

**Figure 2:**
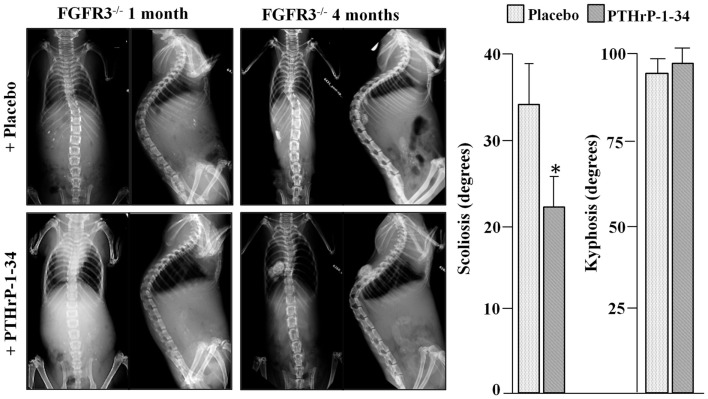
**Treatment with PTHrP-1-34 inhibits scoliosis but not kyphosis in FGFR3^−/−^ mice: At 1 month of age, FGFR3-null mice were randomly assigned to groups to receive pellets loaded with placebo (upper, *N* = 18) or with PTHrP-1-34 (lower, *N* = 16), which were placed adjacent to the thoracic spine through a small skin incision**. Coronal (left) and sagittal (right) radiographs were taken at the time of pellet implantation and at monthly intervals until 4 months, when the mice were euthanized and the spines removed for micro CT analyses (graph). Continuous, local treatment of the growing spine with PTHrP-1-34 in FGFR3^−/−^ mice inhibited progression of scoliosis but had little impact on kyphosis *p < 0.03

To analyze scoliosis, cohorts of FGFR3^+/+^ and FGFR3^−/−^ mice were anesthetized at monthly intervals from 1 to 4 months to capture high resolution coronal and sagittal X-rays of the spine. Serial radiographs of representative mice, shown in Figure 1, reveal an established sigmoid curve at 2 months in the FGFR3^−/−^ mouse compared with the FGFR3^+/+^, which increases in severity to 4 months when the mice were euthanized. As shown in Figure 2, continuous release of PTHrP-1-34 from pellets implanted adjacent to the thoracic spine at 1 month of age inhibited progression of scoliosis but did not alter the development of kyphosis in FGFR3^−/−^ mice.

### Micro CT analysis of the scoliotic curve in FGFR3^−/−^ mice

**Figure 3:**
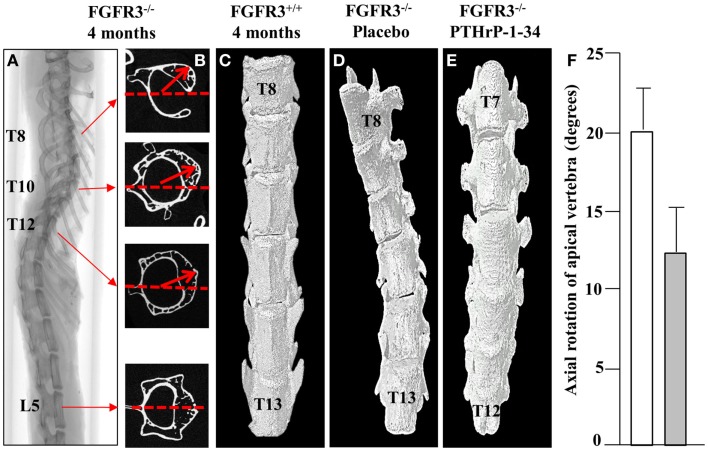
**PTHrP-1-34 inhibits rotation of apical vertebra in FGFR3^−/−^ mice: An X-ray of the intact spine of an FGFR3^−/−^ mouse captured by micro CT (A), is shown adjacent to the mid-region 2D CT images of the identified vertebrae (B)**. The L5 image was aligned in the horizontal axis (dotted line) as a reference point to measure the degree of axial rotation (arrows) on T8, T10, and T12. 3D reconstruction of the lower thoracic spine of FGFR3^+/+^ **(C)** and FGFR3^−/−^ **(D,E)** mice revealed an overall reduction in axial rotation of the vertebrae in FGFR3^−/−^ mice treated with PTHrP-1-34 (C, *N* = 16) compared with mice treated with placebo [**(B)**, *N* = 18]. Quantitative analysis of axial rotation of the apical vertebra in placebo (white) and PTHrP-1-34 (gray) treated mice is shown in the bar graph **(F)**.

In keeping with the clinical assessment of scoliosis, we evaluated the lateral displacement of vertebrae along the horizontal plane and their rotation in the axial plane. Using the L5 vertebra as a reference point outside of the curve, the vertebra with maximal lateral displacement and rotation was identified as the apical vertebra. The 2D cross-sectional micro CT images of a representative FGFR3^−/−^ spine shown in Figure 3 reveal significant rotation of T8 through T12 in the presence of a relatively small lateral displacement of the spine. 3D reconstructions of the lower thoracic spine of representative FGFR3^−/−^ mice show that treatment with placebo (Figure 3B) does not correct the lateral curvature or axial rotation, whereas both are improved in mice treated with pellets that release PTHrP-1-34.

### Treatment with PTHrP-1-34 has minimal impact on vertebral bone

**Figure 4:**
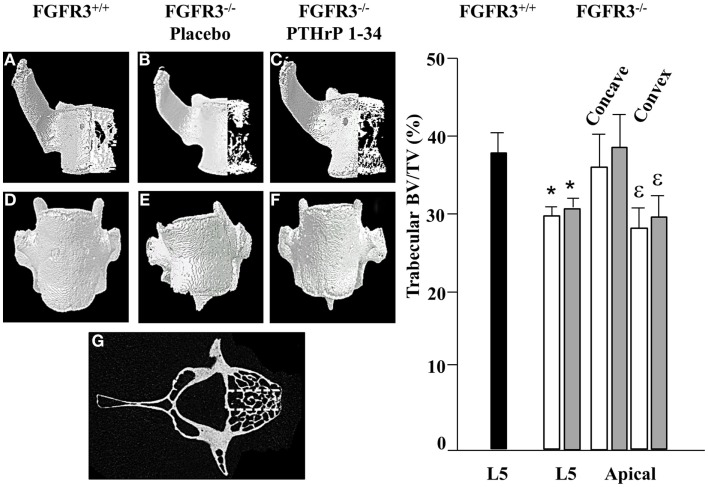
**PTHrP-1-34 treatment does not alter BV/TV in apical vertebra of FGFR3^−/−^ mice: 3D reconstruction of the apical vertebra from treated FGFR3^−/−^ mice were compared with the corresponding vertebra from FGFR3^+/+^ mice**. Coronal 3D images **(A–C)** show reduced trabecular bone in the FGFR3^−/−^ vertebrae **(B,C)** regardless of treatment, compared with the FGFR3^+/+^ vertebra **(A)**. 3D reconstructions **(D–F)** show wedging of the apical vertebra in placebo treated [**(E)**, *N* = 18] mice compared with that of PTHrP-1-34 treated mice [**(F)**, *N* = 16] or FGFR3^+/+^ control [**(D)**, *N* = 5]. Trabecular bone was segmented into three regions [**(G)** dotted lines] to quantify bone volume as a percentage of tissue volume (BV/TV). The middle segment was excluded and the outer segments were analyzed separately to capture the Concave and Convex sides of the apical vertebrae. Quantitative analysis confirmed a significant reduction in BV/TV in the L5 reference vertebra of FGFR3^−/−^ mice treated with placebo (white bar) and PTHrP-1-34 (gray bar) compared with FGFR3^+/+^ (black bar) mice. Concave/convex asymmetry **(E)** was reflected in a lower BV/TV on the convex compared with concave aspect of the apical vertebrae, with no significant impact of PTHrP treatment. *p < 0.05 compared with FGFR3^+/+^; ε *p* < 0.05 compared with Concave aspect.

To further characterize the defects in the spine of FGFR3^−/−^ mice, the composition and architecture of the apical vertebrae were analysed using quantitative micro CT. The 3D reconstructions shown in the coronal plane (Figures 4A–C) in Figure 4 show the reduction in trabecular bone that characterizes the skeletons of FGFR3^−/−^ mice. The coronal images (Figures 4D–F) confirm the left/right symmetry in the FGFR3^+/+^ vertebra and asymmetry and rotation in the FGFR3^−/−^ placebo treated mouse (Figure 4E), which are partially corrected in the PTHrP-1-34 treated animal (Figure 4F). Quantitative analysis of the L5 vertebrae showed a significant reduction in BV/TV in FGFR3^−/−^ mice. Independent analysis of the concave and convex sides of the apical vertebrae showed BV/TV in the concave side to be comparable to that in FGFR3^+/+^ vertebrae, while that in the convex side remained the same as the L5 reference. Table 2 shows significant differences in the quantitative micro CT parameters for the concave and convex sides of the apical vertebrae in FGFR3^−/−^ spines compared with no difference in FGFR3^+/+^ spines. Between-group comparisons confirmed previous observations of an overall increase in the length of vertebrae in FGFR3^−/−^ mice, but also a significant reduction of height on the concave side of IVDs, measured indirectly as the distance between the endplates of adjacent vertebrae.

**Table 2:** **Quantitative micro CT analysis of apical vertebrae in the primary curve**.

Parameter	Units	FGFR3^+/+^	FGFR3^+/+^	FGFR3^−/−^	FGFR3^−/−^
		concave	convex	concave	convex
		*N* = 17	*N* = 17	*N* = 17	*N* = 17
BV/TV	%	15.22 ± 3.41	15.32 ± 3.00^#^	17.11 ± 5.02	11.73 ± 4.74**
Tr.Th.	mm	0.06 ± 0.01	0.06 ± 0.01	0.06 ± 0.01	0.06 ± 0.01
Tr.N.	mm^−1^	2.44 ± 0.47	2.46 ± 0.34^#^	2.71 ± 0.68	1.99 ± 0.70**
Connectivity	mm^−3^	37.12 ± 21.98	38.24 ± 23.68	48.82 ± 17.01	32.53 ± 19.59**
Height	mm	2.06 ± 0.75^##^	2.06 ± 0.76^##^	2.34 ± 0.49	2.47 ± 0.53
IVD width	mm	0.29 ± 0.08^#^	0.29 ± 0.08	0.23 ± 0.08	0.26 ± 0.07*

Significantly different from FGFR3^−/−^; ^#^*p* < 0.05; ^##^,**p < 0.01.

Significantly different from contralateral side; *p < 0.05; **p < 0.01.

### Treatment with PTHrP-1-34 maintains IVD integrity

**Figure 5:**
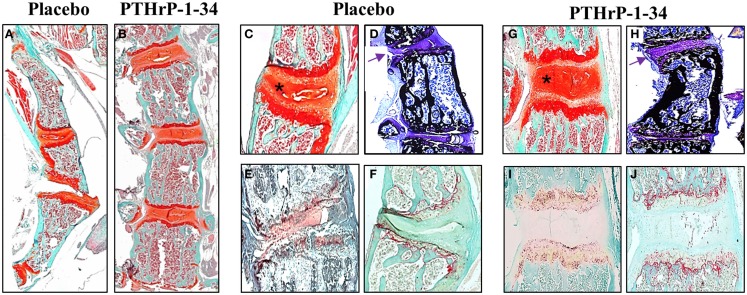
**PTHrP-1-34 treatment inhibits IVD compression in FGFR3^−/−^ mice: After micro CT analyzes representative spines from FGFR3^−/−^ mice were de-calcified, embedded in paraffin, and thin sections stained with Safranin O [(A–C,G) orange] to delineate IVD and cartilage endplates of the vertebra or for ALP (E,I) or TRAP (F,J) enzyme activity**. Some spines were embedded un-decalcified in plastic for von Kossa/Toluidine blue staining of bone **(D,H)**. Histologic analysis of the thoracic spine **(A,B)** confirmed the positive influence of PTHrP-1-34 treatment in reducing curvature and axial rotation shown in the radiology. Higher magnification images of apical vertebrae revealed deterioration and asymmetric compression of the IVD in placebo treated FGFR3^−/−^ mice [asterix **(C)**], which was not apparent in PTHrP-1-34 treated mice. Von Kossa stained sections revealed a concentration of bone on the concave vs convex side of FGFR3^−/−^ vertebrae [**(D,H)** black] and confirmed the correction of the IVD phenotype with PTHrP-1-34 treatment [**(D,H)** arrows]. There were no clear differences in the ALP and TRAP activity in adjacent vertebrae [**(E)** vs **(I)** and **(F)** vs **(J)**].

Qualitative differences in the vertebral bodies and IVDs in response to placebo and PTHrP-1-34 treatment are shown in Figure 5. Low magnification images of Safranin O-stained spines (Figures 5A,B) clearly demonstrated the improvement in linearity in PTHrP-1-34 treated mice. Higher magnification images revealed wedging in association with deterioration of the mucoprotein in the nucleolus pulposus and apparent disorganization of the annulus fibrosus in placebo treated (Figure 5C) compared with PTHrP-1-34 treated (Figure 5G) IVDs. Staining of un-decalcified bones with von Kossa/Toluidine blue (Figures 5D,H) showed more bone on the concave than convex side of the apical vertebra in both treatment groups. However, as shown in the Safranin O-stained sections there was a clear improvement in morphology in PTHrp-1-34 compared with placebo treated mice (arrows; Figure 5D vs Figure 5H). Quantification of ALP (Figures 5E,I) and TRAP (Figures 5F,J) staining in the vertebral end plates also showed no significant differences between placebo and PTHrP-1-34 treated animals (ALP 8.0 ± 7.7 vs 17.3 ± 10.9; TRAP 10.9 ± 4.4 vs 15.6 ± 3.5).

## Discussion

The goal of this study was to analyze the progression of kypho-scoliosis in osteopenic FGFR3^−/−^ mice and to determine if localized treatment with a peptide known to promote bone formation *in vivo* could inhibit progression of the deformity. Micro CT and histological analyses showed that sustained release of PTHrP 1-34 from pellets placed adjacent to the thoracic spine inhibited progression of the scoliotic curve, but had little impact on kyphosis or osteopenia.

A technique for quantification of the lateral displacement of the spine in patients with scoliosis was developed by the orthopedic surgeon John Cobb in the late 1940s and remains as the preferred technique for assessment of curve severity (23). Measurement of the Cobb angle on serial antero–posterior radiographs of the spines of growing mice revealed 62 of 63 FGFR3^−/−^ mice and no FGFR3^+/+^ mice met the criteria for scoliosis at the time of euthanasia between 4 and 6 months of age. Rapid progression of the curve coincided with rapid body growth up to 4 months, when the mice reached skeletal maturity, and slowed thereafter.

The phenotype of FGFR3^−/−^ mice overlaps with that of individuals carrying a missense mutation in the tyrosine kinase domain of FGFR3 that results in camptodactyly, tall stature, and hearing loss or CATSHL syndrome (OMIM 610474) (27, 28). Phenotypes that include scoliosis are also seen in humans and mice with mutations in genes encoding proteins involved in the downstream signaling pathways of cell surface receptors like FGFR3. One example is von Recklinghausen Disease or Neurofibromatosis Type 1. This autosomal dominant disorder arises from disruption of the neurofibromin gene (OMIM 613113), which encodes a large cytoplasmic protein that regulates several intracellular signaling molecules (29). Another example is disruption of the protein tyrosine phosphatase non-receptor type 11 gene (*PTPN11* OMIM 176876) encoding the Src-homology 2 phosphatase downstream of FGF receptors (30).

In contrast to the relatively rare incidence of CATSHL syndrome caused by FGFR3 inactivation, activating mutations in FGFR3 that result in achondroplasia (31) and hypochondroplasia (32) occur at an estimated frequency between 1/15,000 and 1/40,000 live births. Affected individuals exhibit kyphoscoliosis in association with short-limbed dwarfism in the growing post-natal skeleton (33). Others less fortunate carry activating mutations that results in a peri-natal lethal form of short-limbed dwarfism called Thanatophoric dysplasia (TD) (34). Like the FGFR3^−/−^ mice, these babies show little evidence of kyphoscoliosis at birth. The sporadic mutations that result in TD are distributed throughout the extracellular and intracellular components of FGFR3, which exemplifies the heterogenous nature of the disorder. Targeted mutations in the mouse FGFR3 gene have replicated the phenotype of some human skeletal dysplasias to some extent but have failed to clarify the pathogenesis of scoliotic curve progression (35–37). The consistency with which FGFR3^−/−^ mice develop kyphoscoliosis during post-natal growth identify them as a valuable resource to further explore its pathogenesis and potential therapeutic options.

In comparison with placebo, treatment of FGFR3^−/−^ mice with PTHrP 1-34 appeared to reduce spine curvature suggesting its activity might be complementary to that of FGF signaling through FGFR3 in skeletally mature mice. Previous work showed compound mutant FGFR3^−/−/^PTHrP^−/−^ mice die in the perinatal period with skeletal abnormalities less severe than those seen in the single mutant PTHrP^−/−^ littermates (38). This study concluded that PTHrP regulated the pool of growth plate chondrocytes, while signaling through FGFR3 played a more pronounced role in turnover of cartilage to bone at the chondro–osseous junction. Additional studies in skeletally mature FGFR3^−/−^ mice revealed osteopenia arising from defective osteoblast mineralization (11). Taken together this work supports the hypothesis that complementary signaling through PTH1R and FGFR3 pathways regulate cartilage and bone growth and metabolism by targeting different cell populations.

Recombinant PTH 1-34 is the only anabolic medication for the treatment of osteoporosis that has been approved for use in humans (39), although its mechanism of action remains poorly defined. The results of decades of intense investigation has determined that pre-osteoblasts in bone marrow, mature osteoblasts adjacent to the bone surface, and osteocytes embedded in bone matrix all respond to intermittent stimulation with amino-terminal fragments of PTH and PTHrP (17, 40, 41). In the current study, PTHrP-1-34 treatment resulted in blunting of the coronal plane scoliosis in FGFR3^−/−^ mice as evidenced by a reduction in the discrepancy between concave and convex lengths of the apical vertebra. Rotation of the apical vertebra was also less pronounced in the mice treated with PTHrP-1-34 compared with those receiving placebo. Although the cause of vertebral rotation remains un-defined, there is general agreement amongst physicians treating patients with scoliosis that the degree of rotation of affected vertebra is increased concomitant with curve severity. These changes resulted in less severe scoliotic deformity and closer approximation to the spinal morphology of WT mice, but with little change in the kyphotic phenotype. One possible explanation is that PTHrP-1-34 released from the pellet in the vicinity of the thoraco-lumbar spine promoted the differentiation of osteogenic cells to strengthen the vertebrae. This conjecture is supported by an apparent increase in ALP activity in the vertebral endplates in our study. Further support comes from a systematic search of the literature that suggested intermittent treatment of osteoporosis with PTH 1-34 could improve both the speed of healing and composition of the tissue in cases of spinal fusion (42).

In contrast to the improved symmetry of the apical vertebra and spine in general in FGFR3^−/−^ mice treated with PTHrP-1-34, there was little evidence of an improvement in BV/TV in the apical vertebra compared with the reference L5 vertebra, which was outside of the curve. The higher BV/TV in the concave compared with convex side of the apical vertebra could have resulted from an anabolic response to increased biomechanical strain on the concave side (43). Alternatively, it could result from collapse of the disorganized trabecular network under increased strain, similar to the spine compression fractures seen in patients with severe osteoporosis (44). Given the known biological action of amino terminal PTH and PTHrP on growth plate and articular chondrocytes, the reduction in curvature could also have resulted from the peptide altering growth and/or metabolism of the cartilaginous IVDs. This hypothesis was supported by the reduction in wedging of the IVDs adjacent to the apical vertebra in FGFR3^−/−^ mice treated with PTHrP-1-34 compared with placebo. However, it has been proposed in the literature that IVD wedging occurs secondary to progression of the kyphoscoliotic curve in mice with targeted disruption of the *MECOM* gene, which encodes a transcriptional regulatory protein (45). The lack of apparent impact of PTHrP-1-34 treatment on BV/TV might also reflect inadequacies in the dose and/or release kinetics of the peptide from the pellet. Suffice to say the precise mechanism by which the positive influence of PTHrP-1-34 in correcting progressive scoliosis in growing FGFR3^−/−^ mice awaits additional immunochemical analyses *in vivo* and detailed analysis of intact spines subjected to controlled loading *ex vivo*.

## Author Contributions

CG: planning, data collection, data analysis, manuscript preparation, and analysis. BC: data collection, data analysis, and manuscript preparation. MS: data collection, data analysis, and manuscript preparation. JH: data collection and data analysis. JO: planning and discussion. JH: planning, data collection, data analysis, manuscript preparation, and analysis. NS: planning, data analysis, manuscript preparation, and analysis.

## Conflict of Interest Statement

The authors declare that the research was conducted in the absence of any commercial or financial relationships that could be construed as a potential conflict of interest.
